# Atypical presentation of Schwannoma mimicking squamous cell carcinoma^☆^

**DOI:** 10.1016/j.abd.2024.07.011

**Published:** 2025-01-22

**Authors:** Pedro Rolo de Matos, Miguel Silva, Gilberto Rosa, Pedro Canão, Filomena Azevedo

**Affiliations:** a Department of Dermatology and Venereology, ULS São João, Porto, Portugal; b Department of Pathology, ULS São João, Porto, Portugal

Dear Editor,

Schwannomas are rare, encapsulated, benign tumors originating from the nerve sheath. Although they often occur as solitary lesions in 90% of cases, they may arise in association with central nervous system tumors in 5% of cases. They may also be a manifestation of type 2 neurofibromatosis (3%) or appear as multiple lesions (schwanomatosis).^1^, ^2^ Schwannomas can occur anywhere in the body along the course of a cranial, spinal or peripheral nerve.^3^

Cutaneous schwannomas (CS) appear as deep dermal or subcutaneous nodular lesions. More rarely, they may be located in the superficial dermis. Clinically, they are characterized as well-circumscribed, skin-colored, firm nodules which are generally asymptomatic. However, when pain or tenderness is present, it is usually associated with compression of the adjacent structures, so paresthesia is confined to the tumor site or radiating along the nerve of origin. In fact, pain, tenderness, or paresthesia may accompany up to one-third of the cutaneous manifestations.^4^ CS most often occurs in the 4^th^ and 5^th^ decades of life, without significant evidence of gender predilection.^5^

A 50-year-old man was evaluated due to a painless lesion on the anterior aspect of the right leg evolving for 2 years, with accentuated growth in the 2 months previous to observation, with ulceration. He denied other symptoms such as pain or paraesthesia or a history of cardiac, pulmonary, or neurological pathology.

An ulcerated nodular lesion with 3 cm in diameter was observed in that location (Fig. 1). The diagnostic hypothesis of squamous cell carcinoma or keratoacanthoma was raised. Excision of the lesion was performed.

**Fig. 1:**
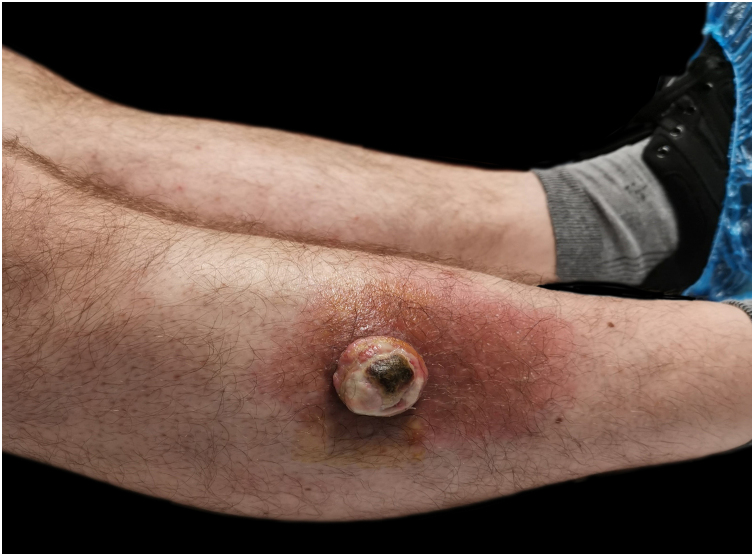
A nodular ulcerated lesion on the lateral aspect of the right leg.

Microscopic examination showed a well-defined tumoral lesion covered by a sclero-hyaline capsule, with ulceration and necrosis of the overlying epidermis and dermis (Fig. 2A and B). The lesion consisted of two patterns: more compacted areas composed of ovoid to spindle-shaped cells with eosinophilic cytoplasm and indistinct cell boundaries (Antoni A pattern) with occasional nuclear palisading (Verocay bodies), and others areas more loosed and hypocellular consisting of cells with clear cytoplasm and well-defined boundaries, with collagenous stroma with myxoid areas and hyalinized wall vessels (Fig. 2C).

**Fig. 2:**
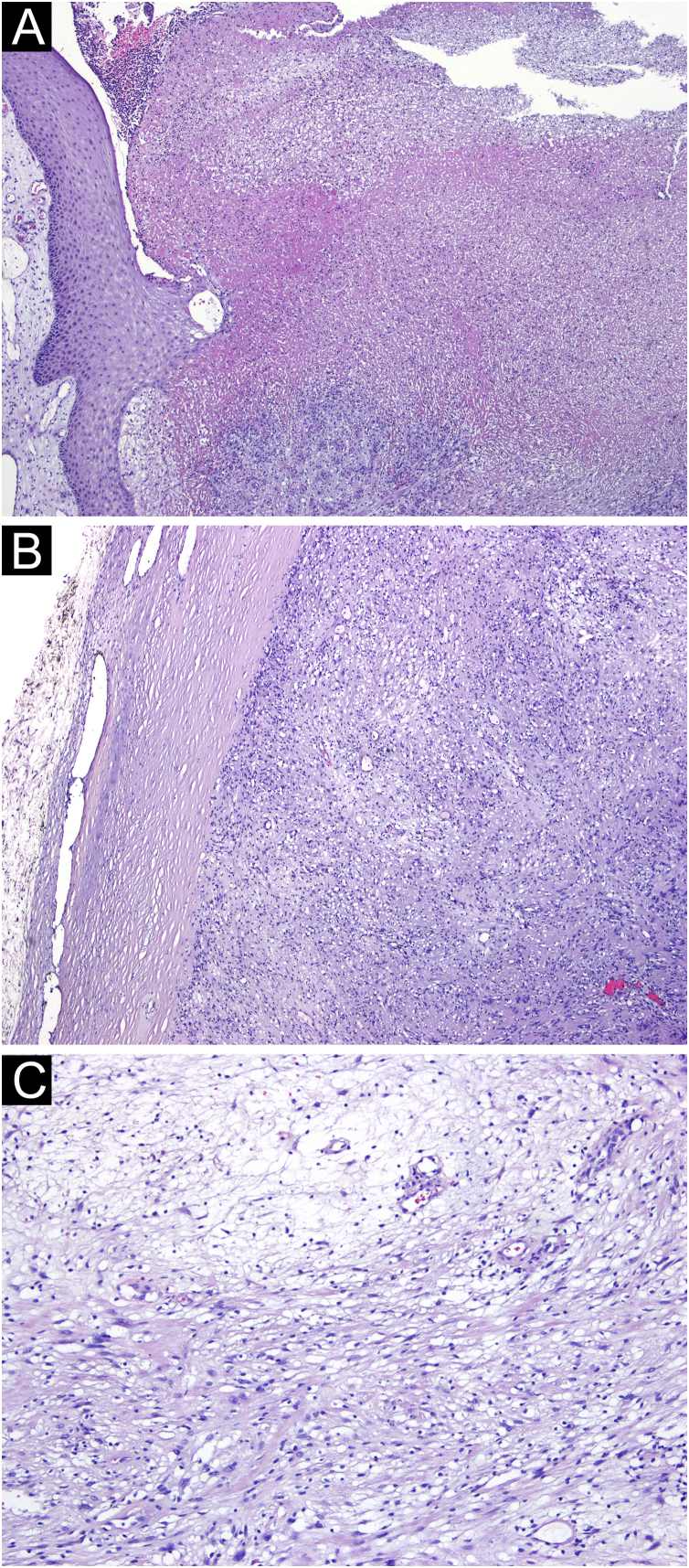
(A) Ulceration and necrosis with suppuration of the adjacent epidermis and dermis (Hematoxylin & eosin, ×40). (B) The lesion was well-delimited by a sclero-hyaline capsule (Hematoxylin & eosin, ×40). (C) Tumoral lesion consisting of ovoid to spindle-shaped cells, with areas of eosinophilic cytoplasm and indistinct boundaries (Antoni A pattern) alternating with more loose and hypocellular areas of cells with clear cytoplasm and well-defined boundaries (Antoni B pattern) (Hematoxylin & eosin, ×100).

In the immunohistochemical study, diffuse expression of protein S100 and SOX-10 was observed, in the absence of HBM45, Melan-A, EMA, among others (Fig. 3A and B). These aspects were suggestive of schwannoma.

**Fig. 3:**
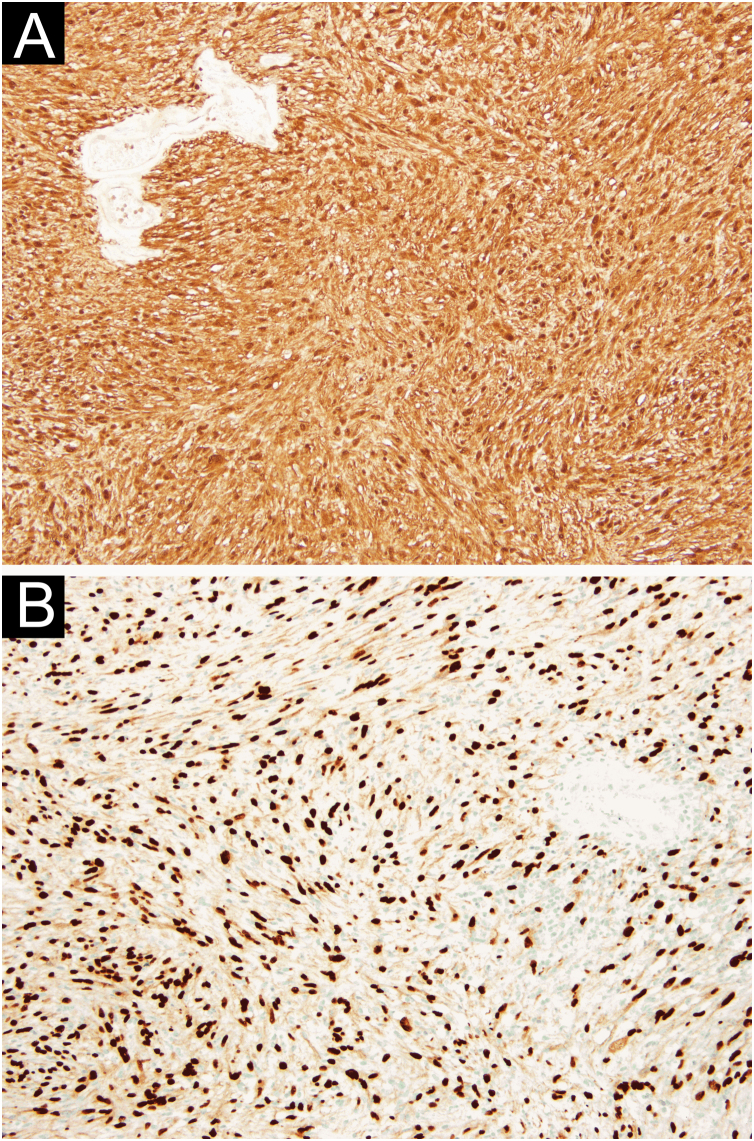
(A) Diffuse expression of S100 protein (×100). (B) Diffuse expression of SOX-10 (×100).

CS is the most common benign peripheral nerve sheath tumor, although occurrence on the lower limbs represents only about 1% of all cases.^6^

Histologically, CS is characterized by two types of histological patterns typically encapsulated by perineurium: Antoni type A and Antoni type B. Antoni A is a highly ordered cellular pattern in which spindle cells are arranged in compact fascicles and their nuclei are disposed of in palisades. Verocay bodies are a characteristic feature in this type of pattern, with collagen matrix arranged into palisading. Antoni type B tissue exhibits a looser structure of mucinous matrix and is less cellular.^5^

The differential diagnosis of CS includes proliferating pilomatricoma, lipoma, desmoid tumor, and epithelial cysts, among others. If tumors of the skin are tender or painful, nine tumors should be considered: leiomyoma, eccrine spiradenoma, neuroma, dermatofibroma, angiolipoma, neurilemmoma (schwannoma), endometrioma, glomus tumor, and granular cell tumor (LEND AN EGG - acronym).^7^

The histological differential diagnosis includes palisaded and encapsulated neuroma (PEN) and neurofibroma.^8^

It is important to appropriately distinguish superficially located schwannoma from PEN, because PEN is encapsulated, located in the upper dermis, and the patterns of interlacing fascicles can be similar to the Antoni type A pattern of schwannoma. Even tough axon-rich PEN does not show typical patterns of Antoni A and B of schwannomas, differentiating between schwannoma and PEN with low or absent axon densities can be troublesome. Neurofibromas are circumscribed but not encapsulated and are composed of spindle cells loosely spaced and wavy collagen strands.^9^

The best treatment option for CS is local excision.^10^

This case corresponds to an atypical presentation considering the rapid growth and ulceration, simulating a malignant lesion, as well as the leg location, which is infrequently described in the literature for schwannoma.

## Authors’ contributions

Pedro Rolo de Matos: Primary author, research, writing.

Miguel Costa Silva: Design, review.

Gilberto Pires Rosa: Support in writing of manuscript.

Pedro Amorso Canão: Pathological analysis and report.

Filomena Moreira Azevedo: Final review.

## Financial support

None declared.

## Conflicts of interest

None declared.
